# A Preliminary Report on Combined Penoscrotal and Perineal Approach for Placement of Penile Prosthesis with Corporal Fibrosis

**DOI:** 10.1155/2008/524392

**Published:** 2008-11-16

**Authors:** John P. Brusky, Viet Q. Tran, Jocelyn M. Rieder, Sherif R. Aboseif

**Affiliations:** ^1^Department of Urology, Kaiser Permanente-Bellflower Medical Center, 9400 East Rosecrans Avenue, Bellflower, CA 90706, USA; ^2^Department of Urology, Kaiser Permanente-Los Angeles Medical Center, 4867 Sunset Boulevard, Los Angeles, CA 90027, USA

## Abstract

*Purpose*. This paper aims at describing the combined penoscrotal and perineal approach for placement of penile prosthesis in cases of severe corporal fibrosis and scarring. *Materials and methods*. Three patients with extensive corporal fibrosis underwent penile prosthesis placement via combined penoscrotal and perineal approach from 1997 to 2006. Follow-up ranged from 15 to 129 months. *Results*. All patients underwent successful implantation of semirigid penile prosthesis. There were no short- or long-term complications. *Conclusions*. Results on combined penoscrotal and perineal approach to penile prosthetic surgery in this preliminary series of patients suggest that it is a safe technique and increases the chance of successful outcome in the surgical management of severe corporal fibrosis.

## 1. INTRODUCTION

Corporal scarring after infection of a penile prosthesis or priapism greatly increases the difficulty of subsequent prosthesis placement. Fibrosis shortens the penis and can obliterate the cavernosal lumen, preventing easy passage of dilators or prosthetic devices. In the case of extensive scarring, resection or cutting of scar tissue with subsequent reconstruction of the corpora with graft materials is often required. This adds additional complexity and time, and increases the likelihood of complication.

To simplify the procedure, we avoid extensive excision of fibrotic tissue whenever possible. We have found with our preliminary series of three patients that a combined penoscrotal and perineal approach allows for a safe dilation of the corpora, even through densely scarred tissue. Grafting of corporal defects is still possible when necessary.

## 2. PATIENTS AND METHODS

From 1997 to 2006, a total of 3 patients with extensive corporal scarring were treated with placement of semirigid penile prosthesis with a combined penoscrotal and perineal approach. All patients had previous removal of infected penile prosthesis and corporal scarring was anticipated. One patient had a history of three prior implants which were removed for infection. In all patients, extensive corporal fibrosis was encountered preventing easy proximal passage of the Hegar metal dilators. In all cases, we felt that blind passage of the metal dilators was not possible or safe, and that prosthetic implantation would not be possible without a secondary approach. A combined penoscrotal and perineal approach was utilized and successful placement of prostheses was accomplished in all patients.

## 3. DESCRIPTION OF TECHNIQUE

All patients are placed in a low lithotomy position for easy access to the perineum and abdomen. The lower abdomen is prepped in the event that autologous rectus fascia is needed for corporal grafting. An extended 10-minute betadine scrub is utilized and the anus is excluded from the draped field. A foley catheter is placed and the surgeon changes his outer gloves. A longitudinal penoscrotal incision is made, except when circumcision is planned, in which case a subcoronal incision with degloving of the penis is performed. Liberal use of antibiotic irrigating solution is utilized throughout the entire procedure.

Longitudinal corporal incisions are made with cutting current electrocuatery and 2–0 vicryl stay sutures are placed in the cut edges for retraction. Metzenbaum scissors are initially used to gently dilate the corporal space. The corporotomies are extended proximally as necessary. Excision of the corpora is avoided if possible. If the corpora can be dilated easily, then a combined perineal approach is not necessary.

**Figure 1:**
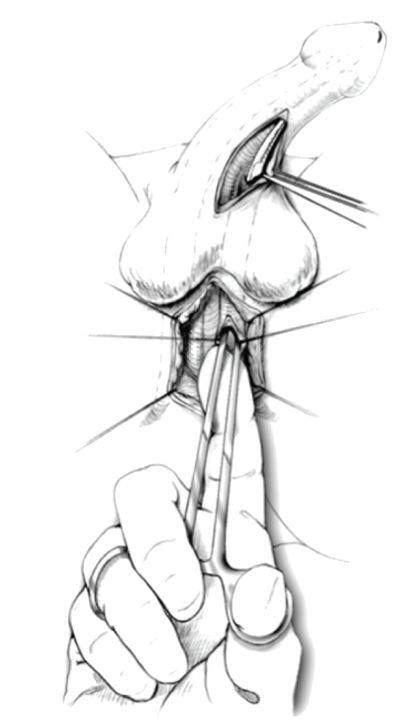
Combined perineal and penoscrotal approach facilitates passage of an instrument from above and below to allow adequate space to be created.

If blind passage of the dilators proximally is felt unsafe, then the perineal approach is also used. A longitudinal perineal incision is performed. The crus of each corpus cavernosum is exposed; a ring retractor with hooks aids with exposure. Longitudinal corporotomies are made and stay sutures are placed. We have found that the proximal corpora are usually less scarred in these cases, and the true lumen is more easily identified. With one finger in the corpora above, a tonsil clamp is passed from below and guided through the area of fibrosis by palpation. The tips of the instrument are pointed away from the urethra to avoid injury (Figure 1). Gentle spreading while withdrawing the instrument helps create the tract. A 6 French ureteral catheter may be placed through the tract to aid in its identification and avoid creation of false passages. Progressively, larger metal dilators are then passed through the tract, either from above or below, whichever proves easier. An appropriately sized prosthesis is then placed. In cases of excessive scarring, we recommend the use of a semirigid prosthesis. The corporotomies are closed with running 2–0 absorbable monofilament suture. If there is excessive tension while closing the corpora over the prosthesis, then a porcine acellular collagen matrix or autologous rectus fascia graft is used to reconstruct the defect.

## 4. RESULTS

Three patients with postinfection fibrosis following prior removal of a penile prosthesis were implanted using the combined penoscrotal and perineal approach. All patients had extensive bilateral corporal fibrosis. Semirigid penile prostheses were placed in all patients. To aid in closing corporal defects, autologous fascia was grafted in two patients. Mean follow-up time was 91 months (range 15 to 129 months). To date there have been no complications and no reoperations.

## 5. DISCUSSION

Penile fibrosis may result from untreated priapism, previous penile prosthesis removal, or intracavernosal injection therapy. Severe fibrosis can greatly complicate the placement of subsequent penile prostheses. The favored approach for severely fibrotic corpora includes excision of fibrotic tissue and grafting with a variety of materials as necessary to repair the defect. Others have advocated corporoscopic resection of fibrotic tissue [1].

In 1986, Herschorn et al. described a two-incision, combined penoscrotal/subcoronal technique for facilitating placement of prostheses in cases of severe distal corporal scarring [2]. Rajpurkar et al. described a minimal scar excision technique through a perineal approach, with a secondary subcoronal incision when necessary for distal scarring [3].

Our approach is somewhat different in that we start with the more familiar penoscrotal approach on all patients. The only initial difference is in patient positioning; a low lithotomy position provides access to the perineum if needed. In most instances when scarring was predicted, however, we were able to safely pass the prostheses proximally without the need for a second incision. In these cases, the low lithotomy position did not interfere with the purely penoscrotal approach.

We find that the most difficult and potentially dangerous step in prosthesis placement with corporal fibrosis is proximal dilation. Blind dilation with excessive force may cause false passages, crural perforation, or urethral injury. The pendulous corpora, however scarred they may be, are more easily visualized and confidently manipulated. Previously, operations have been aborted when proximal dilation was not achieved. Identification of the true corporal lumen is often easier when approached more proximally through a perineal incision. With corporal openings both proximally and distally, a long pointed clamp is more easily passed through the corpora with direct palpation of the instrument tip with the opposite hand. Placing a ureteral catheter through the tract helps maintain accuracy while dilating the tract with gentle spreading of a clamp or metzenbaum scissors. Subsequent passage of metal dilators is then facilitated.

In these difficult cases, we prefer to use semirigid prostheses, although we believe that the same technique can be applied for placement of inflatable prostheses. Summerton et al. used downsized inflatable cylinders as tissue expanders in cases of severe fibrosis, later replacing them with larger cylinders [4]. Regardless of the type of prosthetic placed or the need for corporal excision or grafting, we based our preliminary result that using a combined penoscrotal and perineal approach greatly increases the chance of successful prosthetic placement.

## 6. CONCLUSION

Our preliminary result, based on three patients, suggests that a combined penoscrotal and perineal approach to penile prosthetic surgery is safe and increases the chance of successful outcome in the surgical management of severe corporal fibrosis. We do intend to enroll more patients to confirm these results.
